# Predicting hypertension onset from longitudinal electronic health records with deep learning

**DOI:** 10.1093/jamiaopen/ooac097

**Published:** 2022-11-25

**Authors:** Suparno Datta, Ariane Morassi Sasso, Nina Kiwit, Subhronil Bose, Girish Nadkarni, Riccardo Miotto, Erwin P Böttinger

**Affiliations:** Digital Health Center, Hasso Plattner Institute, University of Potsdam, Potsdam, Germany; Hasso Plattner Institute for Digital Health at Mount Sinai, Icahn School of Medicine at Mount Sinai, New York, New York, USA; Digital Health Center, Hasso Plattner Institute, University of Potsdam, Potsdam, Germany; Hasso Plattner Institute for Digital Health at Mount Sinai, Icahn School of Medicine at Mount Sinai, New York, New York, USA; Digital Health Center, Hasso Plattner Institute, University of Potsdam, Potsdam, Germany; Digital Health Center, Hasso Plattner Institute, University of Potsdam, Potsdam, Germany; Digital Health Center, Hasso Plattner Institute, University of Potsdam, Potsdam, Germany; Hasso Plattner Institute for Digital Health at Mount Sinai, Icahn School of Medicine at Mount Sinai, New York, New York, USA; Department of Medicine, Icahn School of Medicine at Mount Sinai, New York, New York, USA; Hasso Plattner Institute for Digital Health at Mount Sinai, Icahn School of Medicine at Mount Sinai, New York, New York, USA; Department of Genetics and Genomic Sciences, Icahn School of Medicine at Mount Sinai, New York, New York, USA; Digital Health Center, Hasso Plattner Institute, University of Potsdam, Potsdam, Germany; Hasso Plattner Institute for Digital Health at Mount Sinai, Icahn School of Medicine at Mount Sinai, New York, New York, USA; Department of Medicine, Icahn School of Medicine at Mount Sinai, New York, New York, USA; Windreich Department of Artificial Intelligence and Human Health, Icahn School of Medicine at Mount Sinai, New York, New York, USA

**Keywords:** machine learning, electronic health records, deep learning, hypertension

## Abstract

**Objective:**

Hypertension has long been recognized as one of the most important predisposing factors for cardiovascular diseases and mortality. In recent years, machine learning methods have shown potential in diagnostic and predictive approaches in chronic diseases. Electronic health records (EHRs) have emerged as a reliable source of longitudinal data. The aim of this study is to predict the onset of hypertension using modern deep learning (DL) architectures, specifically long short-term memory (LSTM) networks, and longitudinal EHRs.

**Materials and Methods:**

We compare this approach to the best performing models reported from previous works, particularly XGboost, applied to aggregated features. Our work is based on data from 233 895 adult patients from a large health system in the United States. We divided our population into 2 distinct longitudinal datasets based on the diagnosis date. To ensure generalization to unseen data, we trained our models on the first dataset (dataset A “train and validation”) using cross-validation, and then applied the models to a second dataset (dataset B “test”) to assess their performance. We also experimented with 2 different time-windows before the onset of hypertension and evaluated the impact on model performance.

**Results:**

With the LSTM network, we were able to achieve an area under the receiver operating characteristic curve value of 0.98 in the “train and validation” dataset A and 0.94 in the “test” dataset B for a prediction time window of 1 year. Lipid disorders, type 2 diabetes, and renal disorders are found to be associated with incident hypertension.

**Conclusion:**

These findings show that DL models based on temporal EHR data can improve the identification of patients at high risk of hypertension and corresponding driving factors. In the long term, this work may support identifying individuals who are at high risk for developing hypertension and facilitate earlier intervention to prevent the future development of hypertension.

## INTRODUCTION

High blood pressure (hypertension) is considered the leading risk factor for deaths worldwide, among major risk factors, such as smoking, alcohol use, and low physical activity.^1^ As of 2015, hypertension had a global prevalence of 22.1% (24.1% in men and 20.1% in women)^2^ and, due to its asymptomatic nature, it was named by the World Health Organization (WHO) as the “silent killer.”^3^ Every available means and data sources should be utilized to assess for hypertension proactively and establish its diagnosis and treatment as early as possible. In recent years, secondary use of electronic health record (EHR) data have shown to play a major role in improving research-based management of chronic diseases.^4^ In particular, temporal data residing in EHRs, tracking major clinical aspects of a patient’s health status over time, combined with state-of-the-art machine learning (ML) and deep learning (DL) methods hold the potential to improve early detection and management of chronic conditions, such as hypertension or diabetes.^5^^,^^6^ In recent years, DL architectures such as convolutional neural networks (CNN) and recurrent neural networks (RNN) which are able to address the longitudinal nature of EHR data, have demonstrated promising results for a variety of clinical prediction problems. A CNN architecture called Deepr (Deep record) presented by Nguyen et al^28^ was able to outperform logistic regression for the prediction of unplanned readmission after discharge. An RNN-based architecture called RETAIN (REverse Time AttentIoN) presented by Choi et al^27^ demonstrated better performance than logistic regression and multilayer perceptron for the prediction of heart failure. Given the rapid development in this field, we point the readers to a comprehensive review which summarizes the state of the field and identifies avenues of future deep EHR research.^29^

Several statistical models to predict the risk of hypertension have been reported.^7^ However, they were generally developed using specific and small study cohorts which were mostly prospective. Thus, retrospective and fully data-driven studies in large-scale real-world EHR databases are still scarce. Additionally, most of these models are regression-based (eg, logistic, Cox, Weibull). It should also be noted that while elevated blood pressure at baseline increases the risk of hypertension onset in the next years, higher predictive performance is usually achieved when more clinical features are used. Given the large amount of data in EHR databases, there is still the potential to tap into promising ML and DL approaches, which might lead to more robust and generalizable results.

Ye et al^5^ and Kanegae et al^8^ recently showed that tree-based algorithms can obtain promising results in predicting hypertension with windows of up to 2 years before the onset in different populations. They both used XGBoost and achieved good performances in their validation cohorts, with area under the receiver operating characteristic curve (AUROC) scores of 0.87 and 0.88, respectively (for more information, please refer to Supplementary Table III, Appendix I). However, these studies aggregated features from longitudinal data without preserving sequential information available in EHR databases.

In this article, we introduce a framework based on DL to predict the onset of hypertension from longitudinal EHRs. In particular, the numerical clinical data such as laboratory values and vital signs are first processed using a long short-term memory (LSTM) network while preserving sequential information. The output is then augmented with categorical and binary data, such as demographics, diagnosis, and medications, and the combined representation is pushed through a fully connected layer for the final prediction. We evaluated this model on EHRs from 233 895 adult patients from the Mount Sinai Health System in New York City, United States. Results showed promising predictive capabilities within different time windows before the onset (1 and 2 years). In addition, we report improvements when compared with other methods from the literature based on XGBoost, LightGBM, and logistic regression applied to aggregated clinical features. Our architecture is explainable and aims to leverage temporal aspects of EHRs to characterize hypertension risk. The main goal of our work is to predict the onset of hypertension using an individual’s clinical data and history, which is captured in the EHR system. The presented predictive modeling approach may assist in identification of patients at high risk of developing hypertension in clinical settings, and guide early interventions and preventive measures.

## METHODS

This section presents an overview of the study, including the population and the corresponding clinical descriptors, as well as the ML pipeline implemented to predict the risk of hypertension onset from EHRs.

### Dataset

We used de-identified EHRs from the Mount Sinai Health System (MSHS) data warehouse; the study was approved under IRB-19-02369 by the Program for the Protection of Human Subjects at the Icahn School of Medicine at Mount Sinai. MSHS is a large and diverse urban health system in New York City, NY, which generates a high volume of structured, semi-structured, and unstructured data from inpatient, outpatient, and emergency room visits. As the Mount Sinai Health System (MSHS) includes both hospitals and outpatient facilities, the data collected provides an integrated representation of a patient’s medical history. We used a version of the data containing 4.5 million patients, spanning the years from 1980 to 2018. For each patient, we aggregated general demographic details (ie, age, sex, and race) and clinical descriptors. We included ICD-9 diagnosis codes, medication (RxNorm codes), CPT-4 procedure codes, vital signs, and lab tests (LOINC codes). ICD-10 codes were mapped back to the corresponding ICD-9 versions.

### Definition of hypertension

We defined hypertension in the EHR using the automated electronic phenotyping algorithm previously reported by Nadkarni et al,^9^ which was already validated in the MSHS and 2 other academic health systems of the electronic medical records and genomics (eMERGE) network, including the Marshfield Clinic Research Foundation and Columbia University Medical Center. This blood pressure measurements from the MSHS EHR system has been used in a number of large scale research studies.^31^^,^^32^ The phenotyping algorithm combines 3 criteria that are used to diagnose hypertension:

1. Blood pressure measurements (BPVitals): The patient must have 2 consecutive blood pressure measurements above 140 for systolic blood pressure (SBP) or 90 for diastolic blood pressure (DBP)^10^ within 180 days of follow-up.^11^
2. ICD code for hypertension (ICD): The patient must have received a diagnosis for hypertension, defined by an ICD-9 or ICD-10 code for hypertension.
3. Medication for hypertension (BPMeds): The patient has a record of medications that are used for the treatment of hypertension.

**Figure 1.**
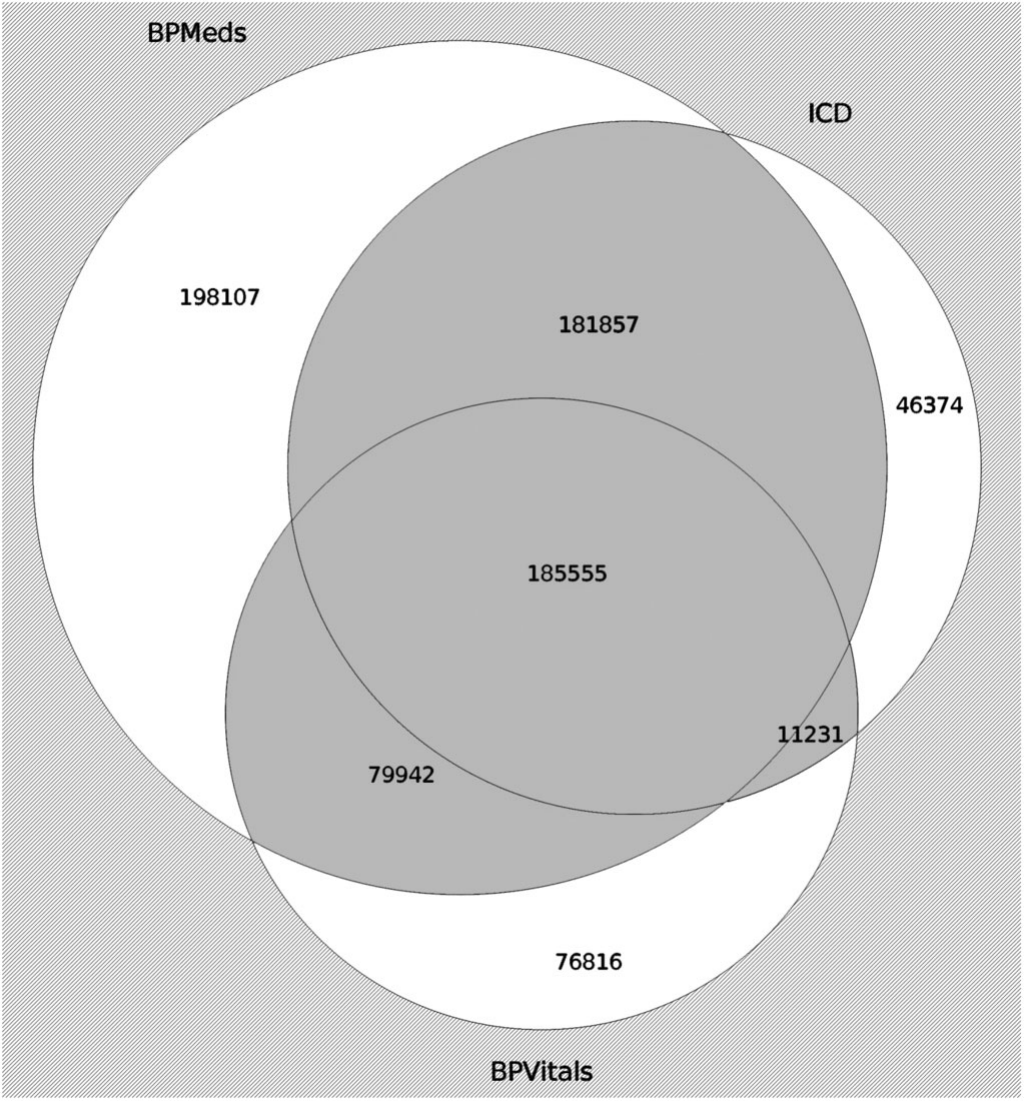
Result of the phenotyping algorithm represented as a Venn diagram. Criterion 1 (BPVitals) represents the presence of higher values of blood pressure within 180 days; criterion 2 (ICD), the presence of an ICD code for hypertension; and criterion 3 (BPMeds), the record of medication for hypertension. The dark grey zone represents the intersection of 2 or more criteria, that is, the selected hypertensive patients (cases). The light grey area outside the circles represents “controls”.

To be part of the case cohort, a patient had to meet at least 2 of these 3 criteria.^9^Figure 1 shows the results of the phenotyping algorithm. The onset of hypertension is defined as the earliest time one of the criteria is met. Previous works such as the one by Ye et al^5^ have only used ICD codes to determine if someone is hypertensive or not. However, as shown by McFarlane et al,^12^ phenotyping algorithms including blood pressure measurements, ICD codes and hypertension medications perform better at identifying hypertensive patients than ICD codes alone. The control cohort was defined as patients who do not meet any of the 3 criteria mentioned above. For the control cohort, the onset date is defined as the last patient encounter date in the dataset. Using a validated phenotyping algorithm allows us to reduce false positives in our case cohort and false negatives in the control cohort.

### Cohorts

**Figure 2.**
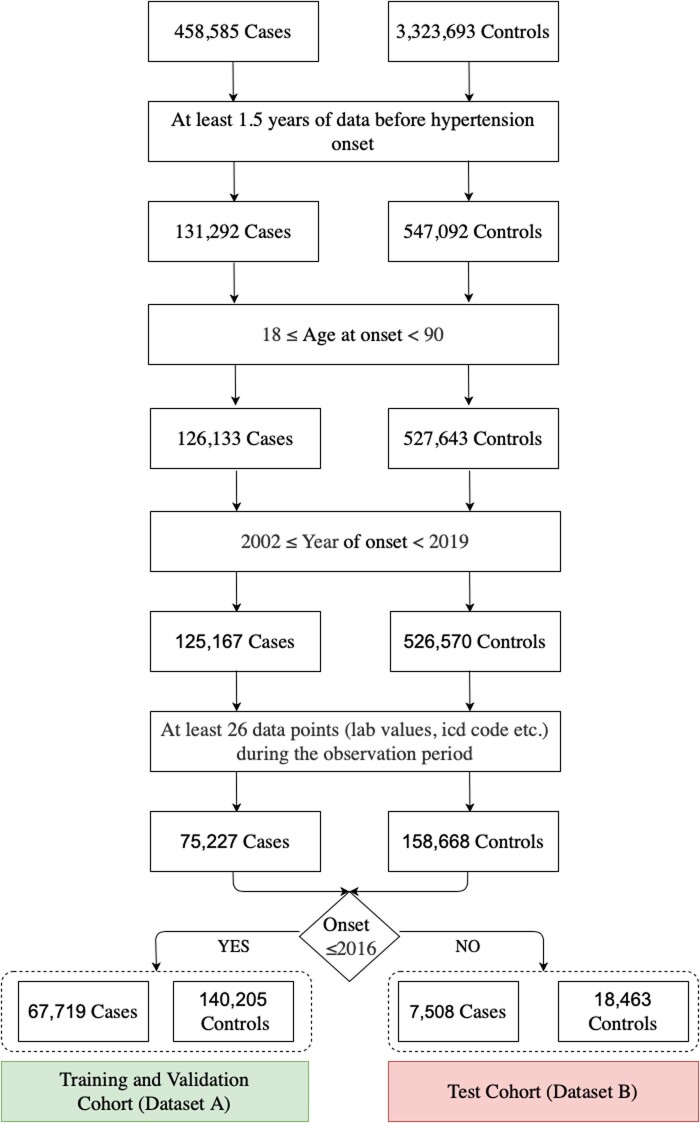
Cohort derivation describing all the inclusion and exclusion criteria.

After identifying the cases and controls from the entire dataset, we applied additional inclusion/exclusion criteria to derive the final cohort (see Figure 2). We filtered out patients that were below 18 and older than 90 years of age. The latter group had to be excluded because their age was not reliably recorded in the database due to privacy protection practices. The year of onset of hypertension was limited between 2002 (systemwide EHR implementation) and 2019 (final year of clinical data warehouse version available for the study). Finally, we applied filters to eliminate records with sparseness of data. To achieve this, we divided the distribution of medical events per patient during the observation window in 4 quartiles. Patients with a number of medical events in the first quartile (ie, lower than 26 events) were excluded. These filters resulted in 233 895 patient records in total; 75 227 of whom developed hypertension (ie, cases) and 158 668 of whom didn’t have any of the 3 case definition criteria (ie, controls). To ensure generalizability to unseen data, we further divided the cohorts into 2 non-overlapping datasets: a training and validation cohort (dataset A) and test cohort (dataset B). Cases and controls in dataset A were related to patients who had an onset date in calendar year 2016 and before. Cases and controls in the dataset B had an onset date in calendar years 2017 and 2018. Table 1 describes the characteristics of the 2 cohorts.

**Table 1.** Description of the dataset A and dataset B Cohort Characteristics

Characteristics	Dataset A		Dataset B	
	Cases	Controls	Cases	Controls
Number of patients	67 719	140 205	7508	18 463
Gender: Male	45.6%	32.4%	43.9%	29.2%
Gender: Female	54.4%	67.6%	56.1%	70.8%
Average age at onset (in years)	58.79	42.57	60.67	43.87
Median number of medical records per patient in the observation period	54	49	58	52

### Feature extraction

We evaluated 2 different strategies to extract clinically relevant features from the patient data. In the first approach, we considered all possible features from the EHR. We extracted all events for patients in case and control cohorts that were recorded before a certain time gap from the hypertension onset date, or the last encounter date for controls. The 2 time gaps we considered in our experiments were 1 and 2 years. The process of determining the observation window for an individual is described in Supplementary Appendix III. All the features that occurred for less than 10% of the patients were discarded. Finally, we selected 74 laboratory values, 11 vital signs and 46 medical procedures. Laboratory values and vital signs were further aggregated with the minimum, maximum and median functions. Additionally, we also considered all demographic features such as age, gender, ethnicity etc. Three different ML models were trained on this aggregated data.

In the second approach, we divided the EHR into 2 types of components, quantitative and categorical. The quantitative components included the laboratory values and vitals, whereas the demographic data, diagnosis, medications, and procedures, were deemed to be categorical. For the quantitative components, the values were divided into bins according to quartiles. Each bin of each medical concept was assigned a unique identifier. Finally, each patient was represented by a “sentence,” which lists the different medical events according to their temporal occurrence. The categorical components were extracted similar to the first approach.

### The temporal data-based approach

**Figure 3.**
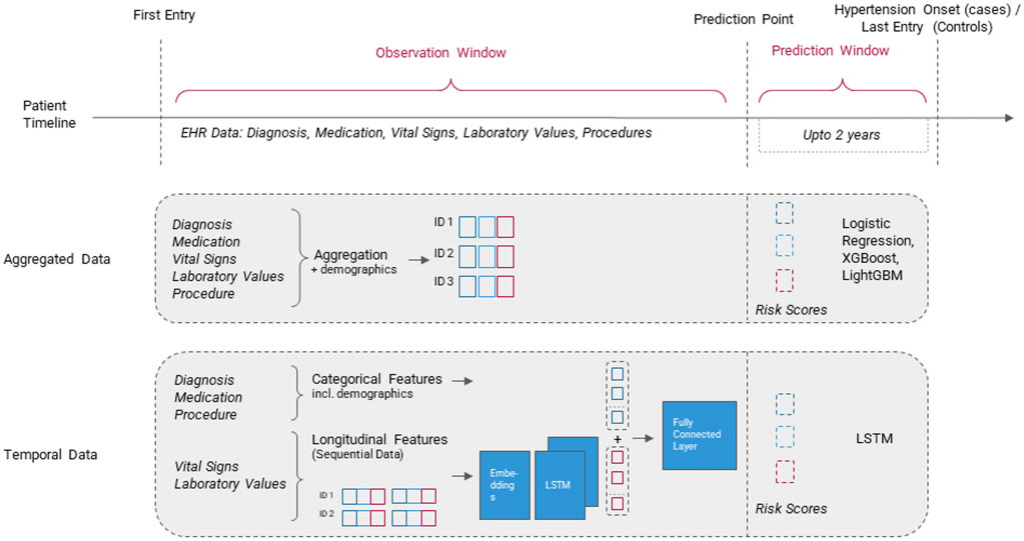
A graphical depiction of the aggregated- and temporal data-based approaches. The aggregated data-based approach uses LR, XGBoost, and LightGBM, whereas the temporal data-based approach employs an LSTM.

The temporal data-based approach uses a DL network to predict the onset of hypertension based on a mix of temporal and aggregated data. The applied DL architecture is summarized in Figure 3 (also in Supplementary Appendix II). At first, the sequence of medical events are brought to equal length either by clipping older events for larger sequences or by padding with 0’s for smaller sequences. Then each sequence is passed to an embedding layer, which outputs a 2D vector with one embedding for each event in the medical concept sequence. It should be noted that these dense embeddings are learned as a part of the supervised network training unlike unsupervised strategies such as word2vec. Each 2D patient vector is then fed into an LSTM layer. LSTMs are RNN, capable of learning long-term dependencies.^13^ The output of the LSTM layer is a vector per patient which is equal in size to the number of units in the LSTM layer. This output is then concatenated with the static features such as diagnosis and demographics, before passing it through a fully connected layer for the final classification. The network design is similar to the one used in Thorsen-Meyer et al.^14^ More details on the network design and hyper-parameters such as layer size, activation function etc. are described in Supplementary Appendix II.

### The aggregated data-based approach

We tested different widely used ML models using bags of medical concepts (ie, disregarding the temporal component) as features. As an initial pre-processing step, we scaled all numerical features to have zero mean and unit variance. All categorical features were encoded with a target encoding, where each instance of a categorical feature is mapped to the probability estimate of the target value. In our case, as we wanted to perform a classification, the numerical representation of the instance corresponds to the posterior probability of the target, conditioned by the value of the categorical attribute.^15^

After those steps, the data went through different imputation strategies. Apart from simple imputation strategies with mean and median, we also used k-nearest neighbor and multivariate imputation by chained equations. It should be noted that some of the ML algorithms we applied, like Extreme Gradient Boosting (XGBoost) and Light Gradient Boosting Machine (LightGBM), can also work without data imputation. These tree-based algorithms usually employ a technique commonly known as sparsity-aware split finding, where the missing data is handled by defining a default direction at each tree node; that is, depending on the feature, a missing value will direct the decision along a left or right path when building the decision tree.^16^ So, we have also defined one approach in which we directly feed the data with missing values into the algorithms without any imputation.

We train the following models on the aggregated data:

1. Logistic Regression: LR works similar to linear regression, but with a binomial response variable. To avoid overfitting we use elastic net regularization.^17^
2. XGBoost: XGboost is a gradient boosted decision tree ensemble algorithm.^16^ To build the decision trees, it makes use of 2 algorithms, namely weighted quantile sketch and sparsity-aware split finding. Notably, XGboost was the best performing model for our work and also for the work of Ye et al.^5^
3. LightGBM: LightGBM, like XGBoost, is a gradient boosted decision tree ensemble algorithm. However, its implementation is quite different and, in many ways, more efficient. The main difference arises in the 2 techniques it uses to handle the creation of splits, namely gradient-based one-side sampling (GOSS) and exclusive feature bundling (EFB). Notably, LightGBM employs leaf-wise tree growth instead of the level-wise tree growth of XGBoost.^18^

The details of the final hyperparameters used for each algorithm can be found in Supplementary Appendix II.

## RESULTS

### Model performance

We compare 2 main approaches for predicting the onset of hypertension based on longitudinal EHR data. In the first approach, longitudinal quantitative traits (ie, laboratory and vital test results) and categorical components (ie, demographic, ICD diagnosis codes, medications, and procedures) of EHR data are fed separately into the proposed DL network. In the second approach, ML models such as XGBoost are trained on aggregated data collected from the EHRs. Both methods are essentially trained on the same data, time span and underlying features. The only difference was that one preserved temporal sequence of quantitative traits and the other only considered the aggregated values.

We evaluated performances based on different metrics. The primary measure that we use to assess the discriminative power of a model was the area under the curve (AUC) of the receiver operating characteristic (ROC) curve, that is, the AUROC. Models with ideal discrimination power have an AUROC of 1.0 and the ones with no discriminatory ability have AUROC equal 0.5. The area under the precision-recall curve (AUPRC) is also reported, since for imbalanced binary classification problems the AUPRC can be more informative than the AUROC.^19^ Moreover, AUPRC is a critical metric for problems like hypertension onset prediction, in which properly classifying the positives is very important. Finally, we also report the F1 score which is the harmonic mean of precision and recall. It should be noted that the F1 score is dependent on the threshold we choose to apply to the probability estimations of the class prediction to get the class labels. Selecting an appropriate decision threshold is highly dependent on the desired outcome of the predictive model (high specificity or high sensitivity). Since our objective was to develop a risk score for hypertension onset, AUROC and AUPRC were considered most relevant.

Table 2 presents results for all model performances and prediction windows, that is, the time period within which we want to predict the hypertension onset. Since the dataset A has also been used as a validation set to tune hyperparameters, we mostly rely on the dataset B performance to assess model performance. For both prediction windows, the DL architecture, that is, the LSTM based model, outperforms all other methods both in dataset A and dataset B. The XGBoost and LightGBM models outperform logistic regression, which is consistent with previous work. The models with a 1 year prediction window perform better than corresponding models with a prediction window of 2 years.

**Table 2.** Model performance comparison for the dataset A (validation) and dataset B (test) reported for 2 different prediction windows of 1 and 2 years before the onset of hypertension

Prediction period	Model	Dataset A			Dataset B		
		AUROC	AUPRC	F1	AUROC	AUPRC	F1
1 year	LR	0.92	0.88	0.86	0.78	0.65	0.61
1 year	XGboost	0.96	0.92	0.90	0.87	0.78	0.77
1 year	LightGBM	0.94	0.90	0.88	0.86	0.78	0.80
1 year	LSTM	**0.98**	**0.96**	**0.93**	**0.94**	**0.87**	**0.80**
2 years	LR	0.92	0.86	0.86	0.77	0.63	0.67
2 years	XGboost	0.95	0.91	0.90	0.84	0.75	**0.77**
2 years	LightGBM	0.96	0.92	0.91	0.84	0.75	0.74
2 years	LSTM	**0.96**	**0.94**	**0.93**	**0.90**	**0.78**	0.71

*Note*: The best performance achieved for each prediction period is marked in bold.

### Model explanation

We used SHapley Additive exPlanations (SHAP) to explain the predictions of all methods. SHAP takes a game theoretic approach to estimate model-agnostic representation of feature importance where the impact of each feature on a particular prediction is represented using Shapley values.^20^ A Shapley value describes, given the current set of feature values, how much a single feature in the context of its interaction with other features contributes to the difference between the actual prediction and the mean prediction. For explaining the tree ensemble models like XGBoost and LightGBM we use the treexplainer module of SHAP.^21^ For the DL model we use the deepexplainer module which uses an algorithm very similar to DeepLIFT to compute feature importance.^22^

**Figure 4.**
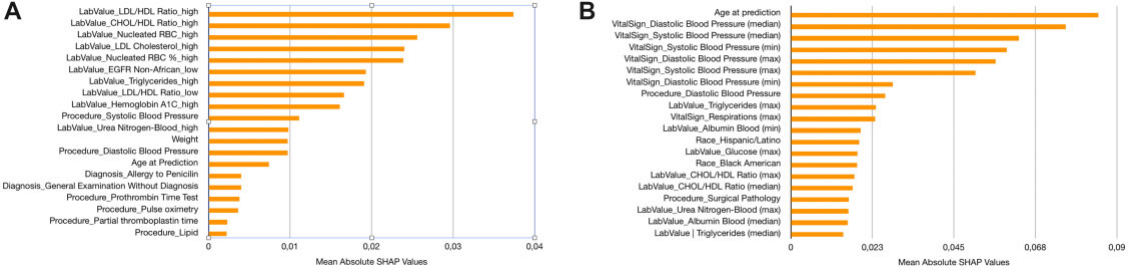
Mean absolute SHAP values of top 20 important features for the prediction window of 1 year for 2 different models. (A) LSTM (deep learning) model feature importance and (B) XGBoost (machine learning) model feature importance.

The top 20 features sorted by their mean absolute SHAP values for the DL and the XGboost model are shown in Figure 4. Higher SHAP values represent a greater impact on model predictions. It should be noted that for the DL model the temporal quantitative components (such as laboratory values and vital signs) were binned into 4 categories, namely high, medium high, medium low, and low based on which quartile the value belonged to. Hence, the mean absolute SHAP value corresponding to “labvalue triglycerides high” indicates what impact the event of having a “high” value of triglyceride had on the prediction on average. In the XGboost model, the quantitative features were aggregated with different aggregation strategies and then fed into the model. Hence, the mean absolute SHAP value corresponding to “labvalue triglycerides (max)” indicates what impact the maximum triglyceride value recorded during the observation period had on the prediction on average. The other 2 aggregation strategies used were median and minimum. The categorical components are delivered to both models in the same fashion.

## DISCUSSION

### Main contributions

In this study, we developed a model for predicting the onset of hypertension within the next 1 or 2 years, based on EHR data. We evaluated different ML and DL algorithms. The models were trained and validated for 207 924 patients then tested on a non-overlapping set of 25 971 patients. In this section, we discuss several important findings with potential implications for clinical care settings.

Firstly, we developed a robust model for predicting the onset of hypertension with promising performance in both the validation and test cohorts. In our experiments, we demonstrated that the DL model built using an LSTM architecture outperforms all other models. With the LSTM-based DL architecture, we achieved AUROCs up to 0.98 in the train and validation cohort (dataset A) and up to 0.94 in the test cohort (dataset B). The better performances can be attributed to the fact that the DL model takes into consideration the temporal nature of EHRs. An LSTM network is a special kind of RNN capable of capturing long-term dependencies from sequential data via gated cells, determining whether or not to maintain information based on the importance it assigns to it. Thus, an LSTM-based model trained on sequential data learns from information about the temporal development of the features unlike models trained on only aggregated data,^14^ which ignore the temporality of clinical data. Contrary to previous work that do not consider the temporal relationships of the EHR data, we show that building models based on temporal data can improve the model performance significantly. In our analysis, we also show that the tree-based ensemble models like XGBoost, LightGBM outperform logistic regression. This can be attributed to the nonlinear relationship between the features which these models can address unlike logistic regression. The models which try to predict hypertension within 1 year of the onset performed marginally better than the ones which tried to predict hypertension within 2 years before the onset. The AUROC for dataset B observed for the 1 year model are characterized by values ranging from 0.94 to 0.78, whereas for the 2 year models the values ranged from 0.90 to 0.77. The AUPRC for the 1 year model ranged between 0.87 and 0.65, whereas for the 2 year model it ranged between 0.78 and 0.63. This slight improvement of performance is expected as the models with a prediction window of 1 year have more input data close to the event of interest (ie, hypertension onset). The overall high AUROC and AUPRC observed for the DL model are promising for future deployment in clinical care settings to identify patients at high risk of developing hypertension within a 1- or 2-year time window. Focusing on shorter prediction intervals based on dependable risk prediction is relevant in clinical practice to mitigate the deleterious effects of delayed detection, diagnosis and treatment of hypertension.^3^

Our framework also provides clinically relevant understanding of the most salient features driving the predictions of the models. SHAP makes our models explainable both in terms of the importance of individual features for hypertension onset in general and also those at the patient level. Focusing on the features that SHAP found to be important for the DL model, we notice that laboratory values indicative of hyperlipidemia play the most important role in the prediction of the onset of hypertension. High low-density lipoprotein (LDL)—high-density lipoprotein (HDL) ratio, high cholesterol-HDL ratio, high total LDL cholesterol, and high triglycerides are all found to play an important role in the onset of hypertension. Hypercholesterolemia has previously been associated with onset of hypertension.^23^^,^^24^ Similarly, reduction in renal function, as indicated by features low estimated glomerular filtration rate and high blood urea nitrogen, has been associated with increase in blood pressure. For the XGboost model, higher values of SBP and DBP were identified as the most important driving factors. It is well noted in previous works that blood pressure increases progressively from the normal range to the hypertensive range over a period of many years. These patients with slightly elevated blood pressure, that is, SBP between 120 and 139 mmHg and/or DBP of 80–89 mmHg are termed to be prehypertensive. So it is expected that a model developed to predict the onset of hypertension, identifies these prehypertensive patients to be at high risk. High hemoglobin A1c and high blood sugar are both found to be positively associated with hypertension. In EHR-based prediction models, it is often noted that the occurrence/frequency of a particular test carries more information than the actual value of the test.^25^ In our models, we can also observe that higher frequencies of SBP or DBP measurements in the observation period influences the model positively. Importantly, although the relative strengths of driving features vary between LSTM (Fig. 4a) and XGBoost (Fig. 4b), there is considerable and consistent overlap among the top 20 features, respectively, with regard to lipid profiles, renal function or metabolic measures, and blood pressure readings. Finally, some of the features found to be salient such as nucleated red blood cell allergy to penicillin cannot be directly explained. They may rise from biases that exist in our data or they are simply surrogates for other health markers. Overall we observe both conventional and novel features in the SHAP diagrams, which underlines the need of more data-driven ML-based approaches to develop actionable prediction models for risk assessment.

### Limitations

Though showing promising results, our study has certain limitations. First, our results are so far derived from data in one large health system in the United States, and demonstration of generalizability will require their external validation in studies at other health systems. However, the features used in this model are commonly occurring medical tests and procedures which should be readily mappable to EHRs of other institutions. Nonetheless, it should also be noted that clinical practice varies from institution to institution, and so patterns learned from a single health system dataset may not be generalizable to other institutions and may necessitate a partial or complete retraining of the model. We are currently working with partners from other institutions for external validation. Additionally, the guidelines and criteria for diagnosing hypertension is also changing over time.^30^ Such a change would require the models to be retrained using the new definition of cases and controls.

We also recognize, that a model predicting the onset of hypertension in the rather short term of 1 or 2 years before the actual onset, may be less meaningful when considering long-term detrimental organ complications of hypertension, such as cardiac, renal or cerebrovascular events. Nevertheless, shorter-term prediction is meaningful to enable timely, early detection and to prevent deleterious consequences of delayed diagnosis and treatment. In future, we would like to extend the same framework to predict hypertension, 5–10 years before the actual onset date. Our models are also affected by the common biases evident in EHR-based studies such as confounding bias, due to a lack of randomization, and for selection bias, due to missing data.^26^ We do acknowledge that healthy individuals with infrequent interaction with the hospital system may be excluded by our selection criteria, whereas individuals with multiple hospital visits and laboratory tests who are more likely to be included in our cohort are probably also the ones who are more likely to develop hypertension and other cardiovascular complications. Infrequent interaction with the health system also limits our ability to determine the precise onset of hypertension. Although the main goal of our work is to predict hypertension before the onset, due the missing data problem in EHRs, it may occur that the model identifies individuals with undiagnosed hypertension as individuals at risk for developing hypertension.

In addition, though SHAP makes our opaque ML models somewhat transparent, it should be kept in mind that interpretation is not necessarily causation. ML models don’t have any underlying causal structure, and their predictions are completely data-driven. The lack of causal structure means the models might behave differently for sub-populations which are under-represented in the training data. Also, not all of the features we found to be important are actionable (eg, age, race and gender). Even when they are actionable, it is not certain if a physician is able to intervene and alter some of the driving biomarkers that could have a positive impact on the hypertension onset. None of the performance metrics we observe can attest if a model prediction can actually bring a favorable change in patient care and outcome. A real impact of such a model can only be gauged via appropriate prospective clinical evaluation.

## CONCLUSION

We developed an explainable LSTM-based DL network to predict the onset of hypertension based on longitudinal EHR data from a large health system in the United States. We compared our model to models suggested in previous work and successfully outperformed them. Our models can predict hypertension up to 2 years before the actual onset. Pending further evaluation, the presented model is promising for deployment in clinical care settings to identify patients at high risk of developing hypertension within the following year. We propose that the presented model indicate potential to support clinical actionability by targeting interventions on modifiable risk factors, such as lipid or metabolic profiles, to prevent or postpone the onset of hypertension, thereby potentially reducing risk for major adverse cardiovascular events, including cardiac deaths.

## FUNDING

Research reported in this article was supported by the Office of Research Infrastructure of the National Institutes of Health under award number S10OD026880. The content is solely the responsibility of the authors and does not necessarily represent the official views of the National Institutes of Health.

## AUTHOR CONTRIBUTIONS

EPB conceived the project. SD created the study design and carried out the experiments. NK and SB also worked on the experiments. SD, NK, and AMS wrote the manuscript with support from RM and EPB. RM, GN, and EPB supervised the project.

## SUPPLEMENTARY MATERIAL

Supplementary material is available at *JAMIA Open* online.

## Supplementary Material

ooac097_Supplementary_Data

## Data Availability

The data used for this study originate from the Mount Sinai Health System (NYC), but restrictions apply to their availability: used under license for the current study, they are not publicly available. Data will be shared upon reasonable request to the corresponding author with permission of the Mount Sinai Health System.
