# Supplementary material for: Predicting hypertension onset from longitudinal electronic health records with deep learning
Source: JAMIA Open. 2022 Nov 25;5(4):ooac097. doi: 10.1093/jamiaopen/ooac097 (PMC9696747; doi:10.1093/jamiaopen/ooac097)
Supplement: ooac097_Supplementary_Data [file ooac097_supplementary_data.docx]

**APPENDIX I: Related work**

In this section we present the two hypertension onset prediction papers based on machine learning methods that are closely related to our work. The details of these works are described in Table III.

| Work | DATASET TRAIN/TEST | TIME WINDOW | FEATURE IMPUTATION | HIGHLIGHTED  FEATURES | RESULTS (TEST AUROC) |
| --- | --- | --- | --- | --- | --- |
| Ye et. al [5] | EHR  n=1,504,437 55%/45% | 1 Year | KNN | Age, Chronic Conditions Mental Disorder Drugs | 0.870 |
| Kanegae et al. [8] | Health Checkup  n=18,258  75%/25% | 2 Years | MEAN (numerical)  MODE  (categorical) | Blood Pressure Body Mass Index Age | 0.876 |

TABLE III: Description of previous works on machine learning based hypertension onset prediction.

**APPENDIX II: Hyperparameters For Different Models**

The final hyperparameters for the different models used are described below:

1. **LR**: Penalty: 'elasticnet', Solver: ‘Stochastic Average Gradient (SAGA)’, Inverse Regularization Strength: 0.0005, Maximum Iterations: 2000, L1 ratio: 0.5
2. **XGBoost**: Maximum Tree Depth: 7, Regression Alpha: 0.5, Regression Lambda: 1, Learning Rate: 0.01, Max Bin Size: 32, Column sample by Tree: 0.20
3. **LightGBM:** Number of Leaves: 10, Maximum Tree Depth: 7, Learning Rate: 0.01, Maximum Bin Size: 32, Regression Alpha 0.5, Regression lambda: 1, Feature Fraction: 0.18
4. **LSTM**: Embedding Dimension: 100, Maximum Sequence Length: 150, optimizer: Adam, Batch Size: 2048, Epochs: 200, Learning Rate: 1e-3, Activation Function: Tanh


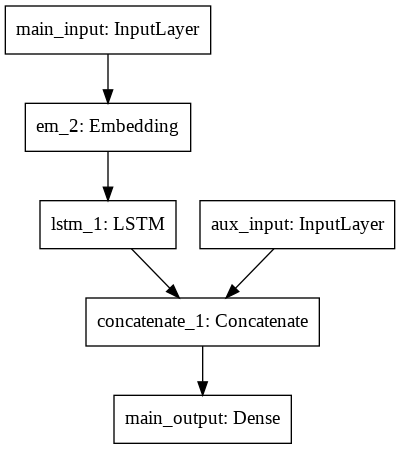


Fig: 5 The architecture of the DL (LSTM) model:

Regarding the runtime for training the different models we made the following observations:

- The training time for the LSTM model (with a three-fold cross validation) was under an hour. We found the runtimes acceptable, especially as we hope models such as this one would not require frequent retraining after being deployed.
- The training of the LSTM model greatly benefited from using a GPU. The particular virtual machine we used for the experiments was a Standard_ND12 from Microsoft Azure. The VM had 24 vCPUs, 224 GB of main memory, 2 GPUs ( Nvidia Tesla P40) with a total of 48GBs of GPU memory.
- Even with the GPU the LSTM model training was still considerably slower than the other ML models.

**APPENDIX III: Observation Window**

In this section we explain the observation window, i.e. the time window within which the features for each individual are observed. The observation window for each individual starts from one or two (depending on the particular model) years before the onset and goes back till the first record of that individual. An individual needs to have at least 26 data records and 1.5 years of observational period to be included in our study cohort (as shown in figure 2). There is no upper limit on the observation window, but records that were older than the 150th record of a particular individual were discarded (only 25% patients from the original cohort had more than 150 data records). The other ML models such as XGBoost received the same data as an input, but instead of sequential data,  here the features were aggregated before. The decision of including patients based on the number of data records (visits) instead of a fixed observation window was done as the interaction with the health system for different individuals over a fixed period of time varied widely. The minimum criteria for the observation window was introduced to make sure the records are reasonably well distributed over time. No upper limit on the observation window was placed as the record distribution over a fixed time window varied widely between individuals. In future, we intend to experiment more with different observation windows and data record lengths to see how it affects the performances of the different models.
